# Silencing of hypothalamic FGF11 prevents diet-induced obesity

**DOI:** 10.1186/s13041-022-00962-3

**Published:** 2022-09-05

**Authors:** Jae Hyun Cho, Kyungchan Kim, Han Chae Cho, Jaemeun Lee, Eun-Kyoung Kim

**Affiliations:** 1 Department of Brain Sciences, Daegu Gyeongbuk Institute of Science and Technology, 333, Techno Jungang-Daero, Hyeonpung-Myeon, Daegu, Dalseonggun 42988 South Korea; 2 Neurometabolomics Research Center, Daegu Gyeongbuk Institute of Science and Technology, 333, Techno Jungang-Daero, Hyeonpung-Myeon, Daegu, Dalseonggun 42988 South Korea

**Keywords:** Fibroblast growth factor 11, Neuropeptide Y, Hypothalamus, Arcuate nucleus, Brown adipose tissue, Thermogenesis, Obesity

## Abstract

Fibroblast growth factor 11 (FGF11) is a member of the intracellular fibroblast growth factor family. Here, we report the central role of FGF11 in the regulation of metabolism. Lentiviral injection of *Fgf11* shRNA into the arcuate nucleus of the mouse hypothalamus decreased weight gain and fat mass, increased brown adipose tissue thermogenesis, and improved glucose and insulin intolerances under high-fat diet conditions. *Fgf11* was expressed in the NPY–expressing neurons, and *Fgf11* knockdown considerably decreased *Npy* expression and projection, leading to increased expression of tyrosine hydroxylase in the paraventricular nucleus. Mechanistically, FGF11 regulated *Npy* gene expression through the glycogen synthase kinase 3–cAMP response element-binding protein pathway. Our study defines the physiological significance of hypothalamic FGF11 in the regulation of metabolism in response to overnutrition such as high-fat diet.

**Supplementary Information:**

The online version contains supplementary material available at 10.1186/s13041-022-00962-3.

## Introduction

The prevalence of obesity and overweight, which are induced by imbalance between energy intake and expenditure, has increased considerably over the decades [1, 2]. The family of fibroblast growth factors (FGFs) consists of 22 members, which are classified into intracellular FGFs (iFGFs), canonical paracrine and autocrine FGFs, and endocrine FGFs depending on their mechanism of action [3, 4]. While canonical and endocrine FGFs are secreted into circulation and act via FGF receptors (FGFRs), iFGFs such as FGF11–FGF14 are acting independently of FGFRs [5, 6]. Mounting evidence suggests that some FGFs have a critical role in the regulation of energy balance including glucose, lipid metabolism, and food intake, suggesting FGFs as therapeutic targets for the treatment of obesity [4]. Central administration of FGF19, endocrine FGF subfamily, reduces the neuronal activity of neurons co-expressing neuropeptide Y (NPY) and agouti-related peptide (NPY/AgRP), thereby improving glucose metabolism and decreasing body weight of mice fed high-fat diet (HFD) [7]. Single intracerebroventricular injection of FGF19 increases glucose disposal rate and ameliorates glucose tolerance in *ob/ob* mice, which lacks gene responsible for the production of leptin and becomes profoundly obese [8]. Adipocyte-specific FGF21, which is endocrine FGF subfamily, knockout mice exhibit the inhibition of white adipose tissue browning in adaptive thermogenesis, and central infusion of FGF21 in obese rats increases insulin-induced suppression of hepatic glucose production and gluconeogenic expression, which in turn increases energy expenditure and insulin sensitivity. [9, 10]. FGF1-knockout mice show aberrant adipose tissue expansion with severe diabetic phenotypes upon HFD feeding, and central administration of FGF1 in wild-type mice suppresses food intake and inhibits FGFR1-containing glucose-sensitive neurons [11, 12]

Recently, it was reported that FGF11 interacts with HIF-1α to induce hypoxia [13], and *Fgf11* knockdown reduces the expression of peroxisome proliferator–activated receptor gamma, thereby inhibiting adipogenesis [14]. However, the function of FGF11, especially its central role in the regulation of whole-body metabolism, remains unknown.

Hypothalamus is one of the most important brain areas that govern metabolism including food intake as well as glucose and energy metabolism [15, 16]. Two major neuronal populations located in the arcuate nucleus of the hypothalamus (ARC) play a fundamental role in regulating metabolism: (i) orexigenic NPY/AgRP co-expressing neurons, which promote anabolism [17, 18], and (ii) anorexigenic neurons co-expressing pro-opiomelanocortin (POMC) and cocaine- and amphetamine-regulated transcript (CART), which induce catabolism [19–21]. These neurons integrate peripheral signals to convey them to the second-order neurons in the paraventricular nucleus (PVN) and deliver multiple signals into their areas of acting such as the nucleus tractus solitarius, dorsal motor nucleus of the vagus, and the ventrolateral medulla in the hindbrain, thereby playing an indispensable role in the regulation of energy metabolism [15, 22, 23]. Numerous studies have demonstrated that NPY/AgRP co-expressing neurons are critical for metabolic functions including food intake, energy expenditure, and thermogenesis [24–28].

Here, we discovered the effect of central *Fgf11* knockdown on multiple parameters involved in whole-body metabolism. The present study contributes to our understanding of the metabolic role of FGF11 in the ARC, highlighting FGF11 as a potential target for the treatment of obesity.

## Materials and methods

### Animal models

Male C57BL/6 mice were purchased from Koatech and housed (one per cage) in individually ventilated cages under a 12-h light/dark cycle (lights on from 7:00 to 19:00) in a temperature- and humidity-controlled room with ad libitum access to water and normal-chow diet (NCD) (LabDiet, Inc., 38057) or HFD (60% kcal from fat; Research Diets, Inc., D12492). Food intake and body weight were observed daily just before the onset of the dark cycle as previously described [29]. The mice were divided into the following groups: control shRNA injected C57BL/6 mice group (shCon); sh*Fgf11-*expressing shRNA injected C57BL/6 mice group (sh*Fgf11*).

### Generation of lentiviruses

Lenti-X 293 T cells (Clontech, 632180) were seeded on 100 mm dishes and transfected with psPAX2 packaging plasmid (6 μg), pMD2.G envelope plasmid (2 μg), and GPIZ constructs (8 μg, green fluorescent protein (GFP), carrying either control (Dharmacon, RHS4346) or *Fgf11*-targeting shRNA using TurboFect (Thermo Scientific, R0531) following the manufacturer’s instructions. To prevent off-target effects, 2 different shRNA plasmids targeting both A and B isoforms of *Fgf11* (Dharmacon, VGM5520-200406248 and VGM5520-200407071) were selected after testing 6 different shRNA plasmids for mouse *Fgf11*. Culture medium containing lentiviruses was harvested and filtered through 0.45 μm syringe filters (Millipore, SLHV033RS) as previously described [29]. To obtain concentrated viruses, 4 successive rounds of ultracentrifugation were carried out in the same ultra-clear centrifuge tubes (Beckman, 344058) at 43,000 × *g* for 120 min at 4 °C [30]. After final centrifugation, pellets were gently resuspended in saline. The titers of lentiviral stocks were determined by flow cytometry [31]; that of control virus was 6.76 × 10^10^ and that of sh*Fgf11*-expressing lentivirus was 6.62 × 10^10^ IU/ml. The concentrated viruses were aliquoted and stored at − 80 °C.

### Stereotaxic surgery

Seven-week-old C57BL/6 mice were acclimated for a week and were anesthetized with 10 ml/kg of body weight of a mixture of Zoletil, Rumpun, and saline 25 min before surgery. Lentiviruses were injected at a speed of 0.5 µl/min (1.32 × 10^8^ IU/2 μl on each side) with a microliter syringe (Hamilton, 7768) using the following coordinates: 1.4 mm posterior to bregma; 6.2 mm ventral; 0.35 mm bilateral targeting the ARC [29].

### Insulin tolerance test (ITT) and glucose tolerance test (GTT)

ITT was conducted 4 weeks after virus injection and GTT 5 weeks after virus injection. Mice were habituated to daily intraperitoneal injections of isotonic saline 3 days before each tolerance test. All procedures were started at 10:00 and performed with reference to general procedures [32, 33]. For ITT, 6 h-fasted mice were injected with 1.0 U insulin/kg (Sigma, Cat#I9278) and blood was collected from the tail vein at the designated times and used to measure glucose with a glucose monitor (Roche, Accu-Chek Active meter). GTT was performed using 16 h-fasted mice by an i.p. injection of 1.5 g/kg glucose (Sigma, Cat#G8270) and blood glucose was assessed as in ITT.

### Determination of brown adipose tissue (BAT) temperature

To evaluate BAT thermogenesis, an infrared camera (FLIR E60, FLIR Systems, Inc.) was used with an intra-red resolution of 320 × 240 pixels. To rule out stress-induced thermogenesis, mice were neglected for an hour while being able to move freely. BAT temperature of each mouse was measured at least 3 times for each round, and the average temperatures from each of 5 rounds were used for analysis.

### Determination of heat generation, O_2_ consumption (VO_2_), CO_2_ production (VCO_2_), respiratory exchange ratio (RER), and total locomotor activity

Two weeks after ARC *Fgf11* knockdown with HFD feeding, indirect calorimetry was performed using metabolic chambers of Comprehensive Lab Animal Monitoring Systems (CLAMS; Columbus Instruments). Mice were housed individually with free access to water and HFD in metabolic chambers Mice were acclimated for 24 h before metabolic assessment. After acclimation, heat generation, VO_2_, VCO_2_, RER, and locomotor activity were measured using an Oxymax system (Columbus Instruments). VO_2_, VCO_2_, and heat production were assessed every 12 min during 24 h and were normalized to body weight; RER was calculated as VCO_2_/VO_2_. Locomotor activity was determined by measuring interruptions in the infrared beams (total X- and Z-beam breaks).

### In situ hybridization

In situ hybridization for the simultaneous detection of *Fgf11* and *Npy* in the ARC was performed using an RNAscope fluorescent multiplex kit (Advanced Cell Diagnostics; ACD). Brains were dissected from three mice, and were rapidly embedded in FSC 22 Frozen Section Media (Lecia, 3801480) and frozen on dry ice. Fresh frozen coronal Sections (20 μm) were cut on a cryostat (Lecia, CM3050S) and dual-labeled for the mRNA of *Fgf11* (ACD, 701) and *Npy* (ACD, 313321-C2) following the manufacturer’s protocol. The *Fgf11* antisense probe targeted the region 888–1891 of the mouse *Fgf11* transcript variant 1 (NM_010198.3), but was cross-reactive with all the other transcript variants (NM_001291104.2, NM_001362623.1, NM_001362624.1). The *Fgf11* sense probe (ACD, 843141) was used as a negative control, and the negative control probe (ACD, 320751) recognizing dihydrodipicolinate reductase, DapB (a bacterial transcript), was also used in parallel with the target probes. Fluorescent in situ hybridization images were taken using an LSM 780 or LSM 800 confocal laser-scanning microscope (Carl Zeiss) with maximal signal separation.

### Immunohistochemistry

The processing, embedding, cryosectioning, and immunofluorescence staining of brain tissue were performed as previously described [34]. The final dilutions of primary antibodies—sheep anti-NPY (1:1000; Abcam, ab6173) and mouse anti-TH (1:1000; Immunostar, 22941)—were 1:1000. The following secondary antibodies were used (both at 1:500): Cy3-conjugated donkey anti-sheep IgG (1:500; Jackson ImmunoResearch, 713–165-147) and AlexaFluor 488–conjugated anti-mouse IgG (1:500; Jackson ImmunoResearch, 715-545-150). Sections were incubated for 5 min at room temperature with 1 μg/mL Hoechst 33342 (Invitrogen, H3570) in phosphate-buffered saline for nuclear staining, mounted on glass slides, and coverslipped with Vectashield Mounting Medium (Vector Laboratories, H-1000). From each mouse (at least 3 mice in total), 3–5 ARC or PVN sections were analyzed using LSM 780 or LSM 800 with maximal signal separation.

### Measurement of immunofluorescence intensity

Standardized settings for image acquisition and processing intensity were performed for relative quantification of NPY and TH fluorescence. To obtain values for NPY immunofluorescence intensity in the ARC and PVN, morphological boundaries of each area were drawn on images. For TH immunofluorescence intensity in the TH neuron, the cell type-specific outlines were plotted with the corresponding gray-scaled TH immunofluorescence images. Single measurements of fluorescence intensity were performed using Image J software (National Institutes of Health, US). NPY fluorescence intensity for each mouse hypothalamic region were averaged from six independent measurements. TH fluorescence intensity for each TH neuron were averaged from four independent measurements. The fluorescence intensity of NPY and TH was plotted using arbitrary units ranging from 0 to 3. NPY-positive axon terminals adjacent within 1.5 µm from TH positive neurons were counted for counting the number of NPY-positive boutons.

### Quantitative RT-PCR analysis of mRNA expression

Total RNA was isolated from cells and tissues using Trizol reagent (Invitrogen, 15596018). The RNA pellet was dissolved in nuclease-free water (Promega, P1193) and total RNA concentration was determined using a NanoDrop spectrophotometer (DeNovix, DS-11). Total RNA, reaction buffer, and GoScript Reverse Transcriptase (Promega, A5004) were mixed in a total volume of 20 μl and reverse transcription was carried out in a thermal cycler (Bio-Rad, C1000) at 25 °C for 5 min, 42 °C for 60 min, and 70 °C for 15 min. Real-time PCR was performed with a SYBR Green PCR kit (TaKaRa Biotechnology, RR820A) in a qPCR machine (Bio-Rad, CFX96) for 40 cycles (95 °C for 10 s, 60 °C for 30 s). The following primers were synthesized by Integrated DNA Technologies: *Cart* Forward, 5′-CGAGAAGAAGTACGGCCAAGTCC-3′; *Cart* Reverse, 5′-GGAATATGGGAACCGAAGGTGG-3′; *Dio2* Forward, 5′-TGCCACCTTCTTGACTTT-3′; *Dio2* Reverse, 5′-GTTTCCGGTGCTTCTTAACC-3′; *Fgf11* Forward, 5′-TCGTCACCAAACTGTTCTGC-3′; *Fgf11* Reverse, 5′-GCCATGTAGTGACCCAGCTT-3′; *Gapdh* Forward, 5′-ATCACTGCCACCCAGAAGAC-3′; *Gapdh* Reverse, 5′-ACACATTGGGGGTAGGAACA-3′; *Npy* Forward, 5′-CAGAAAACGCCCCCAGAA-3′; *Npy* Reverse, 5′-AAAAGTCGGGAGAACAAGTTTCATT -3′; *Pgc1α* Forward, 5′-AGCCGTGACCACTGACAACGAG-3′; *Pgc1α* Reverse, 5′-GCTGCATGGTTCTGAGTGCTAAG-3′; *Pomc* Forward, 5′-GAACAGCCCCTGACTGAAAA-3′; *Pomc* Reverse, 5′-ACGTGGGGGTACACCTTCAC-3′; *Prdm16* Forward, 5′-CCGCTGTGATGAGTGTGATG-3′; *Prdm16* Reverse, 5′-GGACGATCATGTGTTGCTCC-3′. Relative mRNA expression of each target gene was analyzed by the delta-delta Ct method and normalized to that of *Gapdh*.

### Antibodies and chemical reagents

Target proteins were immunoblotted with the following antibodies: phospho-AKT (Ser473; Cell Signaling Technology [CST], 4060), AKT (CST, 9272), β-catenin active (CST, 8814), β-catenin (CST, 9582), phospho-CAMKII (Thr286; CST, 12,716), CAMKII (CST, 3362), phospho-CREB (Ser133; CST, 9198), phospho-CREB (Ser129; MyBioSource, MBS9406211), CREB (CST, 9197), phospho-ERK (Thr202/Thr204, 4370), ERK (CST, 9107), phospho-FOXO1 (Ser256; CST, 9461), FOXO (CST, 2880), GAPDH (CST, 2118), phospho-GSKα and β (Tyr279 and Tyr216; BD Biosciences, 612313), phospho-GSKα and β (Ser21 and Ser9; CST, 8566), GSKα and β (CST, 5676), HA-tag (CST, 3724), phospho-STAT3 (Tyr705; CST, 9145), and STAT3 (CST, 12640). When indicated, cells were treated with 2-deoxy-D-glucose (2DG; Sigma, D6134) to induce glucoprivation; 2DG was dissolved in DPBS (Corning, 21-031-CVR) or saline. 6-Bromoindirubin-3'-oxime (BIO; Sigma, B1686) was dissolved in dimethyl sulfoxide (Sigma, M81802).

### Western blotting

Cells were lysed in 50 mM Tris–HCl, pH 7.4, 250 mM sucrose (Bioshop, SUC507), 5 mM sodium pyrophosphate, 1 mM EDTA, 1 mM EGTA, 1% Triton X-100 (Sigma, T8787), 0.1 mM benzamidine (Sigma, B6506), 1 mM DTT, 0.5 mM PMSF (Sigma, P7626), 50 mM NaF, protease inhibitor cocktail (Calbiochem, 535,140), and phosphatase inhibitor cocktail (Sigma, P5726). Lysates were resolved in SDS–polyacrylamide gels and blotted onto PVDF membranes (Millipore, IPVH00010) for 35 min at 20 V in transfer buffer (25 mM Tris base, pH 7.4, 192 mM glycine, 10% methanol). The membranes were blocked with 5% skim milk for 1 h and incubated with appropriate primary antibodies for 1 h at room temperature or at 4 °C overnight. After 3 washes with TBST buffer (20 mM Tris [Bioshop, TRS001], 125 mM NaCl [Bioshop, SOD001], 0.1% Tween 20 [Sigma, P1379]), each membrane was incubated with appropriate HRP-linked secondary antibody (anti-mouse: CST, 7076S; anti-rabbit: Thermo Scientific, NCI1460KR) and the bands were visualized by using ECL solutions (Thermo Scientific, NCI4080KR; Advansta, K-12045-D50) according to the manufacturer’s instructions. Band intensities were measured and quantified using ImageJ software.

### Cell lines

The embryonic mouse hypothalamic mHypoE-N41 (N41; Cellutions Biosystems Inc., CLU121) and mHypoE-N43/5 (N43/5; Cellutions Biosystems Inc., CLU127) cell lines were maintained in DMEM (Sigma, D5796) with 10% fetal bovine serum (Hyclone Laboratories Inc., SH30919.03) and 1% penicillin/streptomycin (Hyclone Laboratories Inc., SV30010) at 37 °C.

### 2DG and 0-mM glucose treatment

For glucoprivation (2DG) or glucose deprivation (glucose-free), 2DG was added to 25-mM glucose DMEM (Welgene, LM001-07) or 0-mM glucose DMEM (Welgene, LM001-56), was used, respectively. Lenti-X 293T (Clontech, 632180) cells were maintained in DMEM (Hyclone Laboratories Inc., SH30243) under the same conditions as hypothalamic cell lines.

### siRNA transfection

N41 and N43/5 cells were seeded in 6-well plates and transfected with ON-TARGETplus mouse scrambled or *Fgf11* siRNA comprised of 4 different siRNAs. Scrambled siRNAs (100 nM; Dharmacon, D-001810–10-10–05) or *Fgf11* siRNAs (100 nM; Dharmacon, L-045551–01-0010) were transfected using Lipofectamine 3000 for 48 h following the manufacturer’s instructions.

### Statistical analysis

All data were reported as means ± standard error of the mean (SEM). Statistical significance was determined by two-tailed *t-*test or two-way analysis of variance (ANOVA) followed by a Tukey multiple comparison test using GraphPad Prism 8; *p* values < 0.05 were considered statistically significant.

## Results

### Hypothalamic ARC *Fgf11* knockdown prevents obesity

**Fig. 1:**
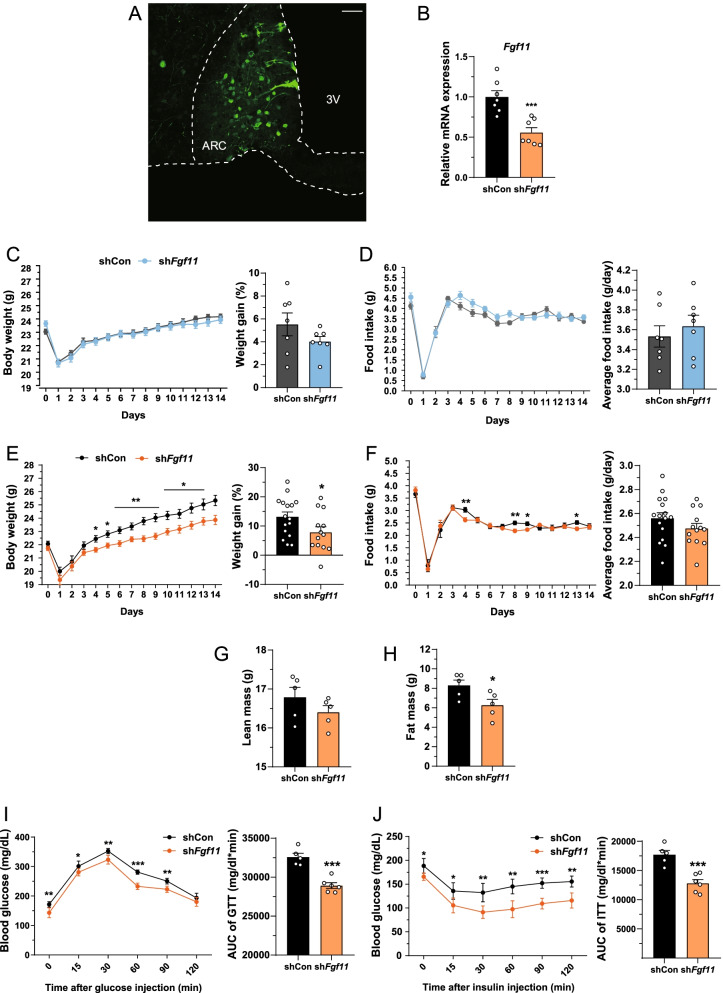
*Fgf11* knockdown in the ARC decreases body weight and fat mass, and improves glucose metabolism in HFD-fed mice. Adult male C57/BL6 mice were fed HFD for 14 days after bilateral injection of lentivirus expressing sh*Fgf11* and GFP into the ARC. **A** Coronal section of ARC. GFP indicates lentiviral infection. **B** mRNA expression of *Fgf11* in micro-dissected ARC sample following injection of sh*Fgf11*-expressing lentivirus. **C** Body weight and weight gain of NCD-fed mice during the experimental period. **D** Food intake and average food intake of NCD-fed mice. **E** Body weight and weight gain of HFD-fed mice during the experimental period. **F** Food intake and average food intake of HFD-fed mice. **G** Lean mass and **H** fat mass. **I** Glucose tolerance test (GTT) and **J** insulin tolerance test (ITT) after lentivirus injection. Data are mean ± SEM; two-tailed *t*-test was used for statistical analysis. *p < 0.05, **p < 0.01 and ***p < 0.001 (non-silencing shRNA control versus sh*Fgf11*). n = 5–16 mice/group

To investigate the central expression of *Fgf11*, we tested *Fgf11* mRNA expression in multiple brain areas and found that it was expressed in the hypothalamus, hippocampus, cortex, and cerebellum (Additional file 1: Fig. S1). Considering that the hypothalamic ARC is a pivotal brain region that governs metabolism [35, 36], we monitored diverse metabolic parameters after the injection of sh*Fgf11*-carrying and GFP-expressing lentivirus into the ARC. Successful ARC lentivirus targeting was confirmed by immunohistochemistry (Fig. 1A). ARC *Fgf11* knockdown was confirmed by a significant decrease in ARC *Fgf11* mRNA level in comparison with non-silencing shRNA control (Fig. 1B). ARC *Fgf11* knockdown had no effect on body weight or food intake of NCD-fed mice (Fig. 1C, D). However, body weight gain of HFD-fed mice was markedly reduced by ARC *Fgf11* knockdown, starting from 4 days after lentivirus injection (Fig. 1E). Food intake of ARC *Fgf11* knockdown mice fed HFD was also decreased transiently after knockdown (Fig. 1F). HFD did not change *Fgf11* mRNA expression levels until 12 weeks but increased them at 16 weeks of HFD (Additional file 1: Fig. S2). While lean mass of ARC *Fgf11* knockdown mice fed HFD remained unchanged, fat mass was substantially reduced (Fig. 1G, H). In GTT and ITT, ARC *Fgf11* knockdown mice fed HFD showed increased glucose clearance rate and insulin sensitivity as compared with control mice, indicating that ARC *Fgf11* knockdown improved the overall systemic glucose homeostasis under HFD conditions (Fig. 1I, J). Altogether, these results suggest that ARC *Fgf11* knockdown prevents obesity including overweight, increase in adiposity, and attenuation of glucose metabolism.

### ARC *Fgf11* knockdown enhances BAT thermogenesis in HFD-fed mice

**Fig. 2:**
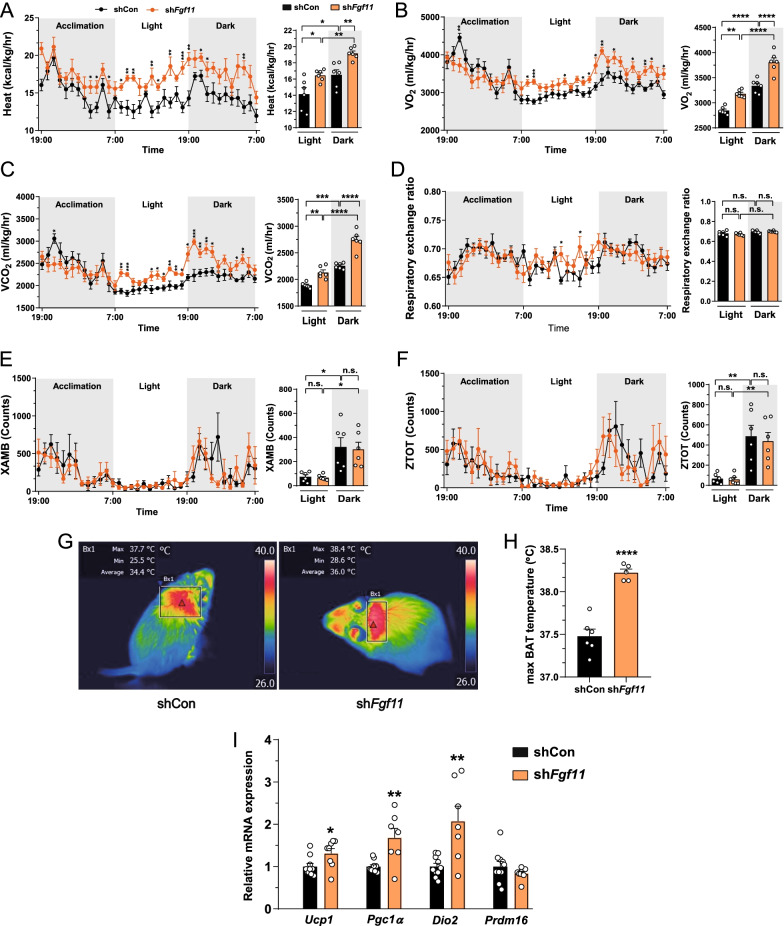
ARC *Fgf11* knockdown increases heat generation, VO_2_, VCO_2_, and BAT thermogenesis without changing locomotor activity. Adult male C57BL/6 mice fed HFD for 2 weeks after bilateral injection of sh*Fgf11*-expressing lentivirus into the ARC. **A**–**F** Heat generation, VO_2_, VCO_2_, RER, total activity at *z*-axis (ZTOT), and ambulatory activity at *x*-axis (XAMB). **G**–**H** Infra-red images of BAT temperature and max BAT temperature on day 14 post-surgery. **I** mRNA expression of 4 representative thermogenic markers on day 14 after ARC *Fgf11* knockdown: Ucp1, Pgc1α, Dio2, Prdm16. *p < 0.05, **p < 0.01, ***p < 0.001, and ****p < 0.0001 (non-silencing shRNA control versus sh*Fgf11*). n = 5–10 mice/group

To evaluate the effect of ARC *Fgf11* knockdown on energy expenditure, we analyzed diverse metabolic parameters including heat generation, O_2_ consumption (VO_2_), CO_2_ production (VCO_2_), respiratory exchange ratio (RER), and locomotor activity using indirect metabolic calorimetry. ARC *Fgf11* knockdown in HFD-fed mice increased heat generation, VO_2_, and VCO_2_ compared with control mice in both light and dark period (Fig. 2A–C). ARC *Fgf11* knockdown did not affect RER or locomotor activity (Fig. 2D–F). Since locomotor activity of HFD-fed mice was not affected by ARC *Fgf11* knockdown, activation of BAT thermogenesis might contribute to the increased heat generation. As expected, BAT temperature of ARC *Fgf11* knockdown mice fed HFD was considerably higher than that of control mice (Fig. 2G, H). Furthermore, *Fgf11* knockdown mice displayed increased mRNA expression of thermogenic genes including *Ucp1*, *Pgc1α*, and *Dio2* compared with control mice (Fig. 2I). Taken together, these data indicate that ARC *Fgf11* knockdown increases heat generation, O_2_ consumption, and CO_2_ production in HFD-fed mice and increases BAT activity by increasing the expression of thermogenic genes.

### Decreased NPY projection from ARC into the PVN caused by ARC *Fgf11* knockdown increases PVN TH expression in HFD-fed mice

**Fig. 3:**
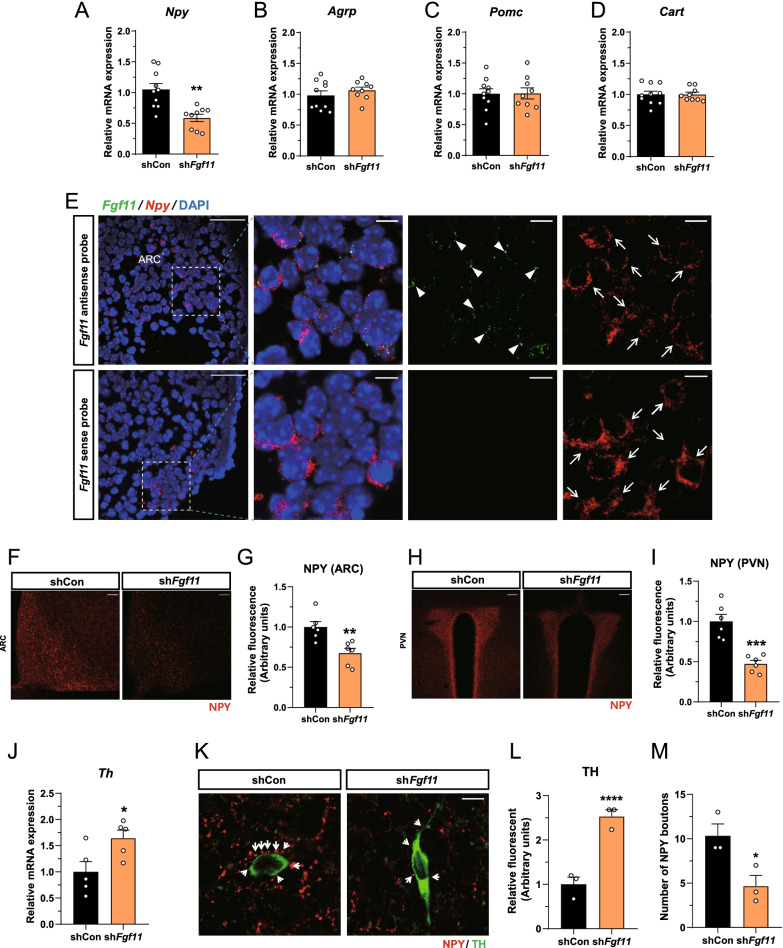
A decrease in NPY expression in the ARC by *Fgf11* knockdown reduces NPY projection into the PVN, increasing PVN TH expression in HFD-fed mice. **A**–**D** Neuropeptide expression in mice fed HFD for 2 weeks after ARC *Fgf11* knockdown. **E** Representative confocal images of in situ hybridization of mRNA of *Fgf11* and *Npy* and corresponding DAPI nuclear counterstaining in the ARC. The *Fgf11* sense probe was used as a negative control. Arrowheads show *Fgf11* mRNA–positive cells. Arrows indicate *Npy* mRNA–positive cells. Scale bar = 20 μm. **F** Coronal sections of the ARC and **G** relative immunofluorescence quantification of ARC of mice fed HFD for 2 weeks after ARC *Fgf11* knockdown. **H** Coronal sections of the PVN and **I** relative immunofluorescence quantification of PVN of mice fed HFD for 2 weeks after ARC *Fgf11* knockdown. Sections were immunostained for NPY; confocal microscopy acquisition settings were the same for both non-silencing shRNA control and sh*Fgf11*. Scale bars **F** 50 μm, **H** 100 μm. **J** *Th* mRNA expression in a micro-dissected sample of the PVN of ARC *Fgf11* knockdown mice fed HFD for 2 weeks. **K** Representative confocal images of double immunostaining for NPY and TH in the PVN 2 weeks after injection of non-silencing shRNA control or *Fgf11* shRNA into the ARC and **L** relative immunofluorescence quantification of TH. Arrows denote NPY-immunoreactive boutons. Scale bar = 5 μm. **M** Number of NPY-positive axon terminals adjacent to TH neuron. *p < 0.05 and **p < 0.01 (non-silencing shRNA control versus sh*Fgf11*). n = 3–10 mice/group

To elucidate the mechanism of increased BAT thermogenesis in ARC *Fgf11* knockdown mice fed HFD, we investigated changes in hypothalamic neuropeptides NPY, AgRP, POMC, and CART, which are related to energy expenditure and thermogenesis [20, 28, 37, 38]. Of these neuropeptides, only *Npy* mRNA expression was significantly decreased by *Fgf11* knockdown in HFD-fed mice (Fig. 3A–D). *Fgf11* knockdown in NCD-fed mice also reduced *Npy* mRNA expression, while other neuropeptides remained unchanged (Additional file 1: Fig. S3). We determined whether FGF11 is expressed in the NPY-expressing neurons. In situ hybridization showed that *Fgf11* mRNA was scattered throughout the ARC, and all *Npy* mRNA–positive neurons contained *Fgf11* mRNA, indicating that *Fgf11* was expressed in NPY neurons (Fig. 3E). NPY immunoreactivity was diminished in both the ARC and PVN of ARC *Fgf11* knockdown mice (Fig 3. F–I). Since ARC NPY overexpression reduces BAT thermogenesis by decreasing TH expression in the PVN [28], we hypothesized that ARC *Fgf11* knockdown activated BAT thermogenesis by inducing PVN TH expression because of a decrease in ARC NPY expression. PVN *Th* mRNA expression was significantly higher in ARC *Fgf11* knockdown mice than in control mice under HFD conditions (Fig. 3J).

Next, to investigate whether this increase in PVN *Th* mRNA expression was a direct consequence of reduced NPY expression caused by ARC *Fgf11* knockdown in the ARC, we conducted immunohistochemistry to identify whether PVN TH neurons were innervated by ARC NPY neurons and were affected by ARC NPY expression. Double immunostaining for NPY and TH showed that PVN TH-positive neurons were in contact with NPY-immunoreactive boutons in their soma and dendrites of mice fed NCD (Additional file 1: Fig. S4), suggesting that PVN TH neurons were innervated by ARC NPY neurons. The immunoreactivity of TH-positive cell bodies in the PVN was increased in *Fgf11* knockdown mice, while the numbers and immunoreactivity of NPY-positive axon terminals adjacent to PVN TH-positive neurons were reduced by ARC *Fgf11* knockdown (Fig. 3K–M). These data demonstrate that ARC *Fgf11* knockdown reduces ARC NPY expression that upregulates PVN TH expression, thereby increasing thermogenesis of BAT.

### FGF11 regulates *Npy* gene expression in NPY-expressing hypothalamic cells

**Fig. 4:**
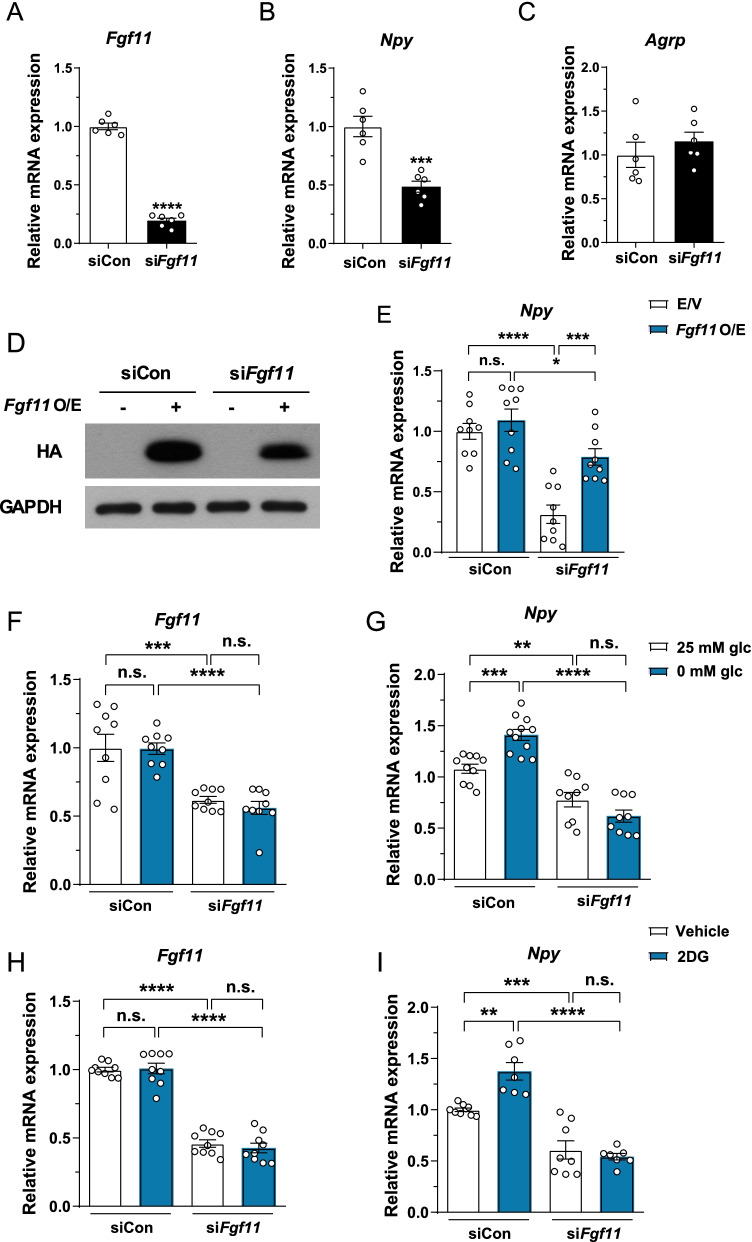
FGF11 regulates *Npy* gene expression. siRNA-mediated *Fgf11* knockdown was conducted in NPY/AgRP co-expressing hypothalamic cells. **A**–**C** mRNA expression of **A** *Fgf11*, **B** *Npy*, and **C** *AgRP* following *Fgf11* knockdown in N41 cells. **D** N41 cells were transfected with empty vector (E/V) or HA-tagged FGF11 and *Fgf11* overexpression and knockdown were confirmed by immunoblotting. **E** *Npy* mRNA expression after *Fgf11* knockdown with *Fgf11* overexpression (O/E). N41 cells were exposed to 0 mM glucose (glc) medium with *Fgf11* knockdown, and mRNA expression of **F** *Fgf11* and **G** *Npy* was measured. N41 cells were treated with 2DG with *Fgf11* knockdown and mRNA expression of **H** *Fgf11* and **I** *Npy* was evaluated. *p < 0.05, **p < 0.01, ***p < 0.001, and ****p < 0.0001, n = 6–11

We further assessed the role of FGF11 in the regulation of *Npy* gene expression in hypothalamic cell line N41 co-expressing NPY and AgRP. *Fgf11* knockdown significantly decreased mRNA expression of *Npy* but not *AgRP* in N41 cells (Fig. 4A–C). On the other hand, *Fgf11* knockdown in N43/5 cells co-expressing *Pomc* and *Cart* did not affect the expression of either gene (Additional file 1: Fig. S5). Of note, overexpression of *Fgf11* rescued *Fgf11* knockdown–induced decrease in *Npy* mRNA expression (Fig. 4D, E ). Since *Npy* expression is induced by low glucose availability [29], we tested whether *Fgf11* knockdown attenuates *Npy* gene induction under low glucose conditions such as glucose-free medium or 2DG treatment. While *Fgf11* mRNA expression was unchanged, *Npy* mRNA expression was considerably increased at both glucose-free medium and 2DG treatment (Fig. 4F–I). Low glucose availability–induced *Npy* gene expression was blunted by *Fgf11* knockdown in glucose-free medium (Fig. 4G), as it was under 2DG treatment (Fig. 4I). In mice, *Fgf11* mRNA expression remained unchanged under fasting condition (Additional file 1: Fig. S6). Taken together, these data indicate that *Fgf11* regulates *Npy* gene expression in hypothalamic N41 cells.

### *Fgf11* knockdown decreases CREB activity but increases GSK3 activity in hypothalamic cells

**Fig. 5:**
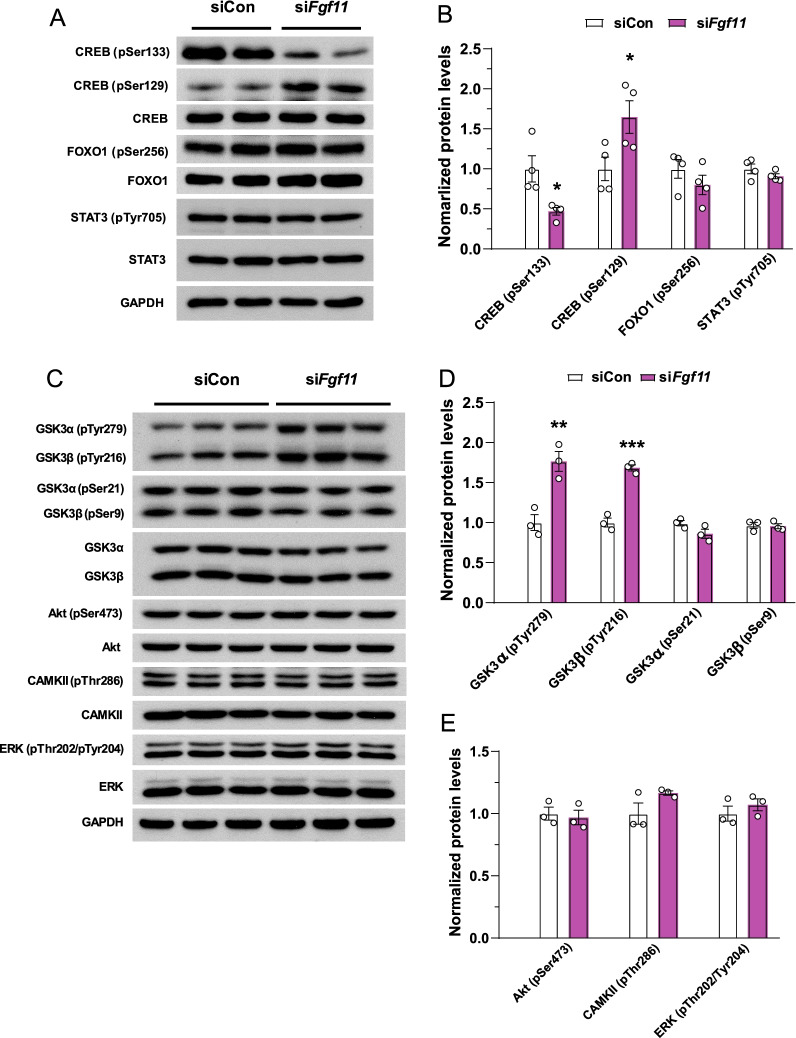
*Fgf11* knockdown in NPY/AgRP co-expressing hypothalamic cells increases CREB and decreases GSK3 activity. N41 cells were transfected with non-silencing siRNA control or si*Fgf11* for 48 h. **A** Western blots of N41 cells: transcription factors of NPY. **B** Quantitation of immunoblots. **C** Western blots of the regulators of CREB activity. **D**, **E** Quantification of immunoblots. *p < 0.05, **p < 0.01, and ***p < 0.001 (non-silencing siRNA control versus si*Fgf11*). n = 3 or 4

To identify the regulatory molecules involved in FGF11-dependent *Npy* gene regulation, we investigated the transcription factors responsible for changes in *Npy* expression following *Fgf11* knockdown. Considering that *Npy* expression is regulated by multiple transcription factors such as CREB, forkhead box protein O1, and signal transducer and activator of transcription 3 [39–43], we assessed the activities of these transcription factors by examining changes in their phosphorylation. Immunoblot analysis showed that none of these transcription factors was affected by *Fgf11* knockdown except for CREB phosphorylation (Fig. 5A, B); phosphorylation of CREB at Ser133 is activatory, whereas that at Ser129 is inhibitory [44–47]. We found that CREB phosphorylation at Ser133 was decreased, while that at Ser129 was increased by *Fgf11* knockdown, indicating that CREB activity was markedly reduced by *Fgf11* knockdown in the hypothalamic cells (Fig. 5A, B). Next, to identify upstream factors responsible for the changes in CREB phosphorylation by *Fgf11* knockdown, we examined the phosphorylation levels of GSK3, protein kinase B, Ca^2+^/calmodulin-dependent protein kinase II, and extracellular signal-regulated kinases since these kinases are known to regulate the activity of CREB [46, 48–52]. Only the phosphorylation of GSK3 at Tyr279 and Tyr216, corresponding to the active forms of GSK3 α and β, respectively, was increased, while that at GSK3 Ser21 and Ser9 was not changed by *Fgf11* knockdown (Fig. 5C, D). Of note, phosphorylation of FYN and PYK2, which are known to control the phosphorylation of GSK3 Tyr216 residue, was unchanged (Additional file 1: Fig. S7) [53]. Phosphorylation of other upstream kinases of CREB was not affected by *Fgf11* knockdown (Fig. 5C and E). These data suggest the possibility that *Fgf11* might regulates CREB activity through the regulation of GSK3 tyrosine phosphorylation.

### *Fgf11* regulates NPY mRNA expression through GSK3-dependent CREB activity

**Fig. 6:**
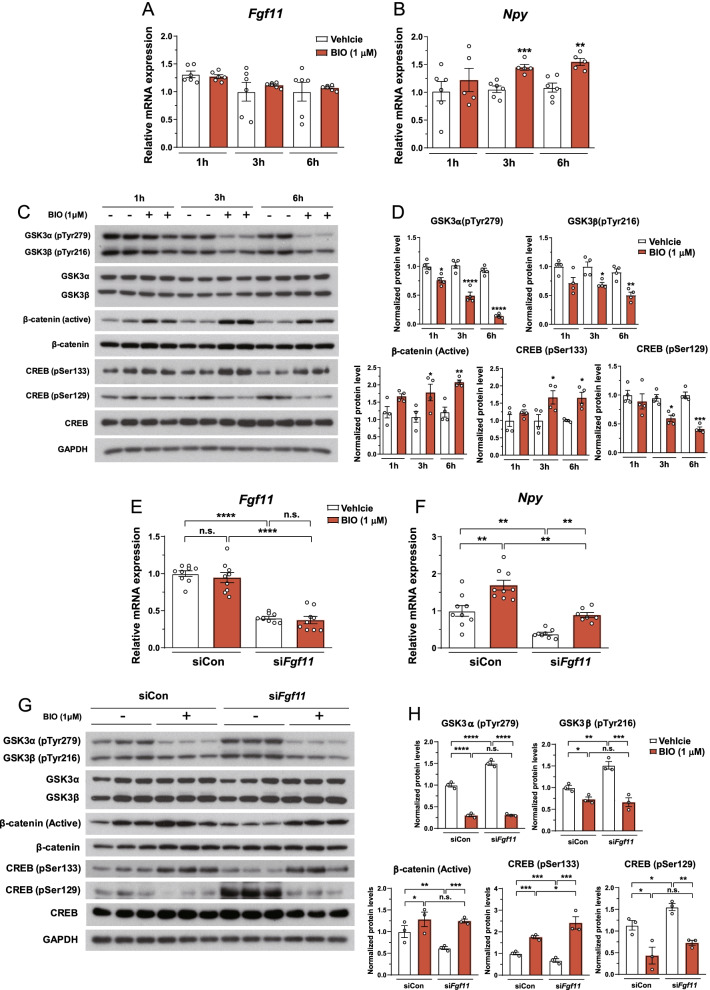
FGF11 regulates *Npy* gene expression via GSK3-dependent CREB regulation in NPY/AgRP co-expressing hypothalamic cells. N41 cells were treated with dimethyl sulfoxide (vehicle) or BIO (1 μM) for 1, 3, or 6 h. **A**, **B** mRNA expression of *Fgf11* and *Npy* after the treatment of BIO. **C**, **D** Western blots of GSK3, β-catenin, and CREB following the treatment of BIO. * *p* < 0.05, ** *p* < 0.01, *** *p* < 0.001, and **** *p* < 0.0001 (vehicle versus BIO). n = 4–6. **E**–**H** N41 cells were transfected with non-silencing siRNA control or si*Fgf11* for 48 h, followed by the treatment with vehicle or BIO for 6 h. **E**, **F** mRNA expression of *Fgf11* and *Npy* after *Fgf11* knockdown with BIO treatment. **G**, **H** Western blots of GSK3, β-catenin, and CREB after *Fgf11* knockdown with BIO treatment. * *p* < 0.05, ** *p* < 0.01, *** *p* < 0.001, and **** *p* < 0.0001, n = 3–9

GSK3α and GSK3β inhibition favors CREB phosphorylation at Ser133 [54]. GSK3β reduces CREB binding activity via the phosphorylation of CREB Ser129 [46, 48, 50]. To determine whether *Fgf11* knockdown reduces CREB activity via GSK3, we treated N41 cells with BIO, a GSK3 inhibitor [55, 56]. BIO treatment did not affect *Fgf11* mRNA expression but increased *Npy* mRNA expression in a time-dependent manner (Fig. 6A, B). GSK3 inhibition by BIO was confirmed by decreased tyrosine phosphorylation and increased accumulation of β-catenin, which is a GSK3 substrate and is degraded by the β-catenin destruction complex [57] (Fig. 6C, D). Additionally, CREB activity was markedly increased by BIO, as demonstrated by increased CREB Ser133 phosphorylation and decreased CREB Ser129 phosphorylation (Fig. 6C, D).

To investigate whether the decrease in *Npy* mRNA expression following *Fgf11* knockdown is due to GSK3-dependent CREB activity, *Fgf11*-knockdown N41 cells were treated with BIO. The inhibition of *Npy* mRNA expression under *Fgf11* knockdown was rescued by BIO (Fig. 6E, F). BIO significantly decreased *Fgf11* knockdown–induced GSK3 tyrosine phosphorylation, as opposed to accumulation of β-catenin (Fig. 6G, H). Importantly, concomitant with the recovery of *Npy* expression, CREB activity decreased by *Fgf11* knockdown was recovered by GSK3 inhibition, as shown by an increase in pSer133 and a decrease in pSer129 levels (Fig. 6G, H). These data demonstrate that FGF11 regulates *Npy* gene expression via GSK3-dependent CREB activity.

## Discussion

FGFs is one of the key players in the regulation of energy balance. However, the function of FGF11 in metabolism remains largely unknown compared with those of other FGFs. Here, we report that hypothalamic FGF11 knockdown improved metabolic features under high-fat conditions.

It has been unknown for the role of FGF11 in regulation of glucose homeostasis. Our study showed that FGF11 knockdown in the hypothalamus improves glucose and insulin intolerance. Previous studies noted that the inhibition of the hypothalamic NPY upstream signaling pathway increases insulin secretion [58, 59] and NPY neuron-specific insulin receptor deficient mice showed impaired glucose homeostasis [60]. Although further study needs to clarify the mechanisms underlying the glucose metabolism and insulin sensitivity regulated by hypothalamic FGF11, it is possible that FGF11 in the NPY neurons could be involved in insulin functions for glucose homeostasis.

Unlike other FGF family such as canonical and endocrine FGFs, the expression of FGF11 family such as FGF12, 13, and 14 is confined to hypothalamic parenchyma including dorsomedial nucleus, ventromedial nucleus, and ARC, not tanycytes [61]. We demonstrated that FGF11 is expressed in the hypothalamus, especially in the NPY neurons in the ARC. This expression pattern reflects the feature of FGF11 family that does not mediate their actions via FGF receptors since FGF receptors in the hypothalamus are mainly expressed in the β-tanycytes [61].

We found that FGF11 regulates *Npy* gene expression by regulating GSK3-dependent CREB activity in the hypothalamus. The activity of GSK3 is mainly controlled by its serine phosphorylation Ser21 in GSK3α and Ser9 in GSK3β [48]. The serine phosphorylation of GSK3 facilitates the action of its N-terminal tail as a pseudosubstrate, hindering binding of primed substrates [62]. GSK3 tyrosine phosphorylation (Tyr 279 in GSK3α and Tyr216 in GSK3β) occurs by auto-phosphorylation during translation and is associated with increased kinase activity [63]. Interestingly, *Fgf11* knockdown did not affect the phosphorylation of GSK3 at serine residue in our study, but changed the phosphorylation at Tyr279 and Tyr216. Therefore, FGF11 might be involved in the regulation of the tyrosine phosphorylation of GSK3α and GSK3β to regulate GSK3 activity.

It has been unknown which physiological conditions regulate the gene expression of *Fgf11*. In our study, we first demonstrated that hypothalamic *Fgf11* gene expression was significantly increased by 16-week-HFD, but not by fasting. According to a previous study, the full manifested features of obesity develop after 16 weeks of HFD [64]. Therefore, the expression of *Fgf11* can be regulated under physiological conditions such as fully developed obesity although it is not clear whether the increase in *Fgf11* gene expression is a cause or result of the development of obesity. Considering the role of hypothalamic FGF11 in the whole-body metabolism, it is likely that the increase in *Fgf11* gene expression is associated with the progression of obesity. Future studies are needed to elucidate the mechanism(s) by which the hypothalamic *Fgf11* is regulated by HFD or other physiological conditions.

Another point is that *Fgf11* knockdown in N41 cells did not change the activity of the kinases such as FYN or PYK2, which is known to regulate the activity of GSK3 [53]. FGF11 is not a kinase [3, 5, 6], and it has an N-terminal nuclear localization signal and directly acts with HIF-1α in the nucleus [13]. Our immunoprecipitation analysis in N41 cells indicates that FGF11 does not directly bind to GSK3 (data not shown). It is possible that FGF11 regulates the tyrosine phosphorylation of GSK3 indirectly. Although further study will be needed to understand how FGF11 regulates the phosphorylation of GSK3 and the related regulatory mechanisms, our results unveil a previously unknown role of FGF11 in the regulation of *Npy* gene expression in the ARC, by which FGF11 regulates BAT thermogenesis under HFD conditions.

ARC *Fgf11* knockdown in mice fed HFD transiently decreased food intake in our study. A significant decrease in *Npy* gene expression by ARC *Fgf11* knockdown contributed to the induction of PVN TH expression, which regulates BAT thermogenesis rather than reduction of food intake. NPY derived from the ARC regulates BAT thermogenesis via a relay of tyrosine hydroxylase in the PVN through the sympathetic output [28]. Accordingly, substantial weight loss caused by the ARC *Fgf11* knockdown may be attributed to increased energy expenditure through BAT thermogenesis.

**Fig. 7:**
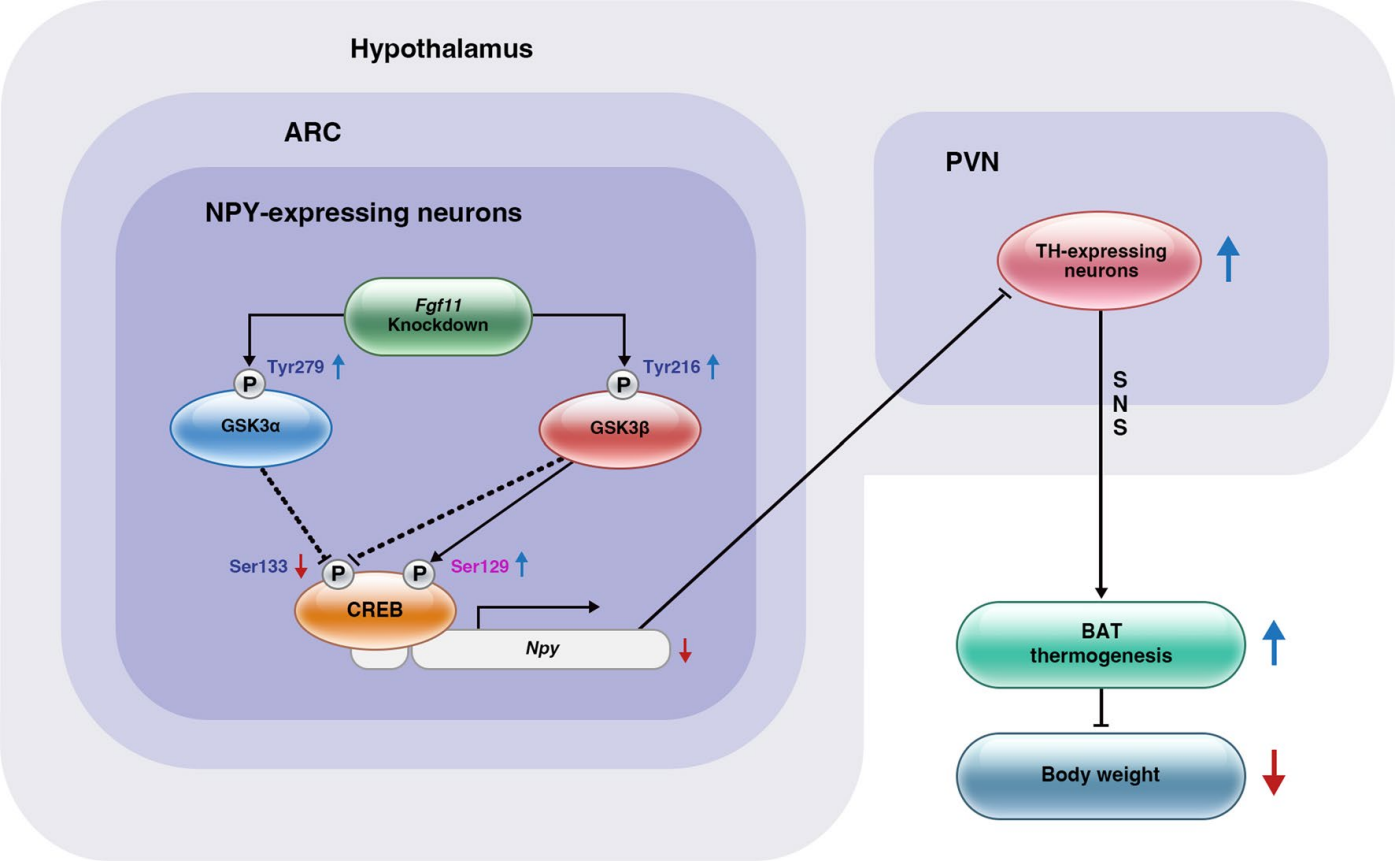
Schematic diagram of *Fgf11* knockdown in prevention of obesity. *Fgf11* knockdown increases tyrosine phosphorylation of GSK3α and GSK3β, thereafter reducing CREB phosphorylation to decrease NPY expression. This results in a reduction of NPY projection into the TH neurons in the PVN, leading to increased TH expression. Consequently, TH induction elevates BAT thermogenesis, reducing body weight

We demonstrated that FGF11 in the ARC plays a role in the energy balance by regulating *Npy* expression and affecting BAT thermogenesis and body weight (Summarized in Fig. 7). FGF11 silencing in the ARC increases BAT thermogenesis and energy expenditure by downregulating *Npy*, which overcomes energy surplus and improves the obese phenotypes under HFD conditions. The delineation of the central role of hypothalamic FGF11 in the regulation of bodily metabolism extends our understanding of the biological functions of FGF11. Overall, our study highlights the importance of FGF11 as a potential therapeutic target for the treatment of obesity.

## Supplementary Information

**Additional file 1: Figure S1.** Central distribution of Fgf11 mRNA. **Figure S2.** Hypothalamic Fgf11 mRNA expression following HFD feeding. **Figure S3. **Effect of Fgf11 knockdown on neuropeptide mRNA expression in the ARC. **Figure S4.** A representative confocal image of double immunostaining for NPY and TH in the PVN of NCD-fed mice. **Figure S5.** Neuropeptide mRNA expression after Fgf11 knockdown in POMC/CART co-expressing cells. **Figure S6.** Effect of fasting on Fgf11 mRNA expression in the hypothalamus. **Figure S7.** Phosphorylation of upstream kinases of GSK3 after Fgf11 knockdown in NPY/AgRP co-expressing cells.

## Data Availability

The datasets used during the current study are available from the corresponding author on reasonable request.

## Abbreviations

2DG: Deoxy-D-glucose
AgRP: Agouti-related peptide
ARC: Arcuate nucleus of the hypothalamus
BAT: Brown adipose tissue
CART: Cocaine- and amphetamine-regulated transcript
CAMKII: Ca^2+^/calmodulin-dependent protein kinase II
CREB: CAMP response element–binding protein
Dio2: Iodothyronine deiodinase 2
ERK: Extracellular signal–regulated kinase
FGF: Fibroblast growth factor
FGFR: Fibroblast growth factor receptor
FOXO1: Forkhead box protein O1
GAPDH: Glyceraldehyde 3-phosphate dehydrogenase
GSK3α: Glycogen synthase kinase-3 alpha
GSK3β: Glycogen synthase kinase-3 beta
GTT: Glucose tolerance test
HA: Human influenza hemagglutinin
HFD: High-fat diet
iFGF: Intracellular FGF
ITT: Insulin tolerance test
NCD: Normal-chow diet
NPY: Neuropeptide Y
Pgc1α: Peroxisome proliferator–activated receptor gamma coactivator 1-alpha
POMC: Pro-opiomelanocortin
Prdm16: PR domain–containing 16
PVN: Paraventricular nucleus of the hypothalamus
STAT3: Signal transducer and activator of transcription 3
Ucp1: Uncoupling protein-1
