# Supplementary material for: Silencing of hypothalamic FGF11 prevents diet-induced obesity
Source: Mol Brain. 2022 Sep 5;15:75. doi: 10.1186/s13041-022-00962-3 (PMC9447329; doi:10.1186/s13041-022-00962-3)
Supplement: Supplementary file 1 — Additional file 1: Figure S1. Central distribution of Fgf11 mRNA. Figure S2. Hypothalamic Fgf11 mRNA expression following HFD feeding. Figure S3. Effect of Fgf11 knockdown on neuropeptide mRNA expression in the ARC. Figure S4. A representative confocal image of double immunostaining for NPY and TH in the PVN of NCD-fed mice. Figure S5. Neuropeptide mRNA expression after Fgf11 knockdown in POMC/CART co-expressing cells. Figure S6. Effect of fasting on Fgf11 mRNA expression in the hypothalamus. Figure S7. Phosphorylation of upstream kinases of GSK3 after Fgf11 knockdown in NPY/AgRP co-expressing cells. [file 13041_2022_962_MOESM1_ESM.pdf]

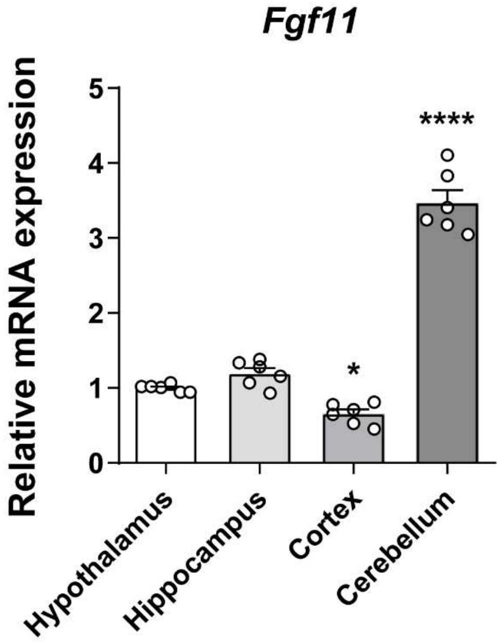

Supplementary Figure 1. Central distribution of *Fgf11* mRNA. Mouse brains were micro-dissected into the hypothalamus, hippocampus, cortex, and cerebellum, and *Fgf11* mRNA expression was analyzed using qRT-PCR in each brain region. * $p < 0.05$, **** $p < 0.0001$ (hypothalamus versus cortex and cerebellum, respectively), n = 6 mice/group.

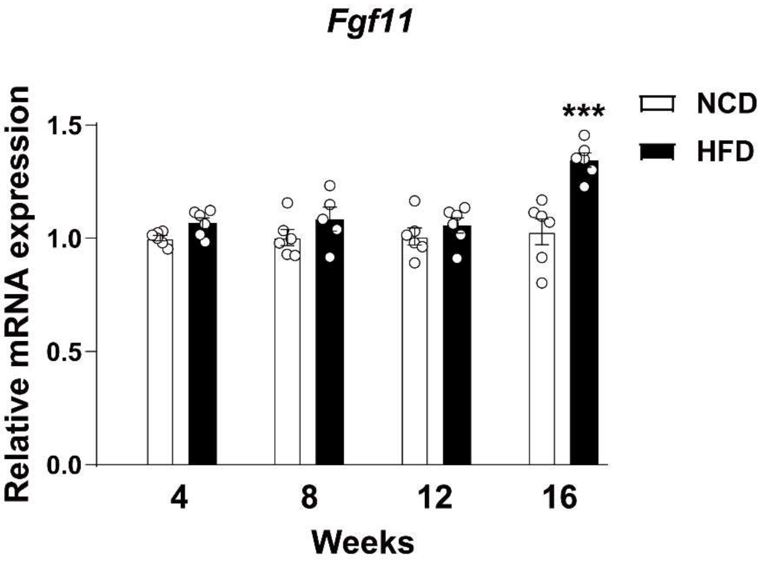

Supplementary Figure 2. Hypothalamic *Fgf11* mRNA expression following HFD feeding. Mice were fed NCD or HFD for 4, 8, 12, and 16 weeks, and hypothalamic expression of *Fgf11* was measured using qRT-PCR. *** $p < 0.001$ (NCD versus HFD), n = 5–6 mice/group.

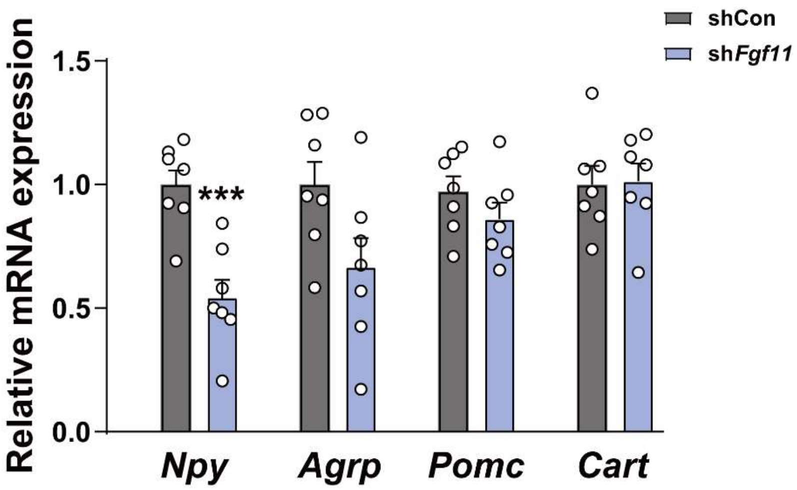

Supplementary Figure 3. Effect of *Fgf11* knockdown on neuropeptide mRNA expression in the ARC. Mice were fed NCD for 14 days after *Fgf11* knockdown and *Npy*, *AgRP*, *Pomc*, and *Cart* mRNA expression was measured using qRT-PCR. *** $p < 0.001$ (non-silencing shRNA control versus sh*Fgf11*). n = 7 mice/group.

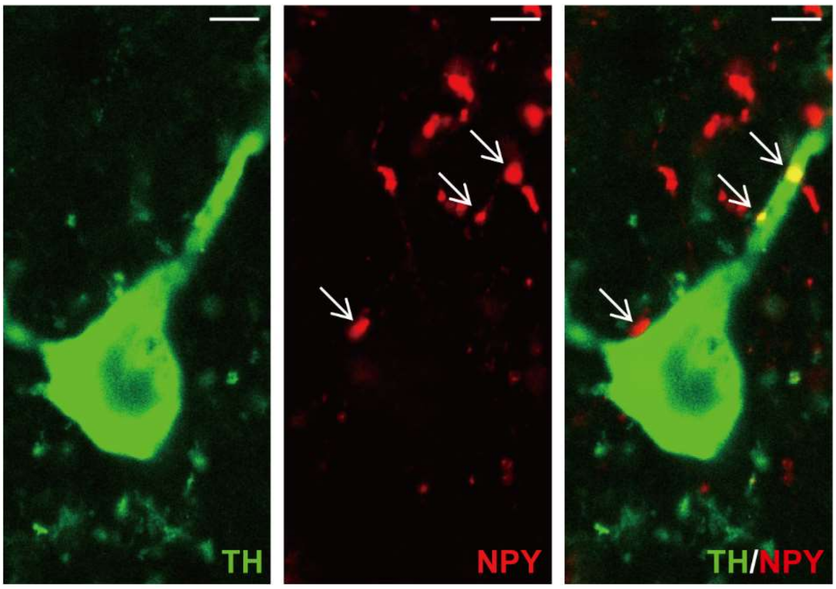

Supplementary Figure 4. A representative confocal image of double immunostaining for NPY and TH in the PVN of NCD-fed mice. Arrows indicate axon terminals of ARC NPY neurons innervated into PVN TH neurons. Scale bars = 5 μm.

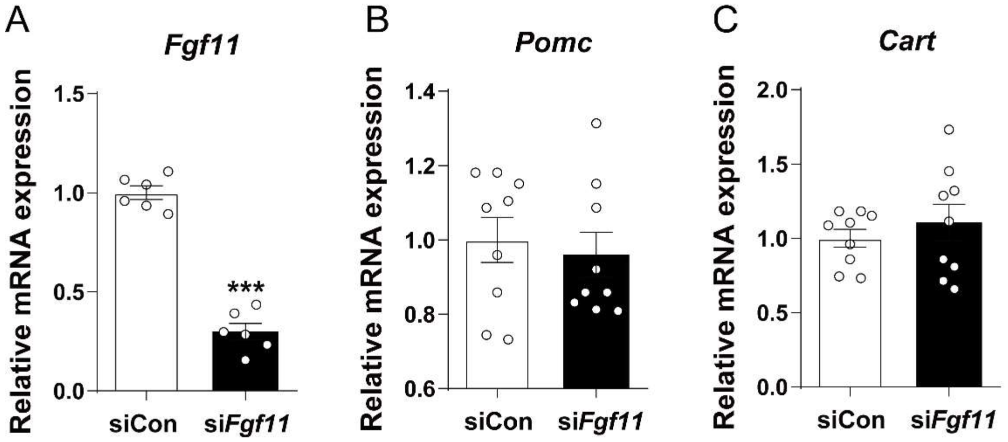

Supplementary Figure 5. Neuropeptide mRNA expression after *Fgf11* knockdown in POMC/CART co-expressing cells. N43 cells were transfected with non-silencing control siRNA or si*Fgf11* and the expression of *Fgf11*, *Pomc*, *Cart* mRNA was analyzed using qRT-PCR *** $p < 0.001$, (control siRNA versus si*Fgf11*), n = 6–9.

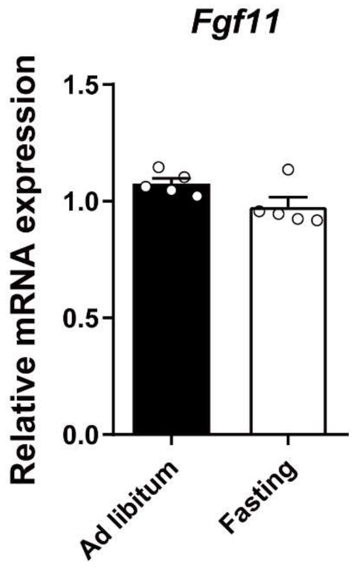

Supplementary Figure 6. Effect of fasting on *Fgf11* mRNA expression in the hypothalamus. Eight-week-old mice were exposed to ad libitum or fasted conditions for 48 h and *Fgf11* mRNA expression was measured using qRT-PCR. n = 5.

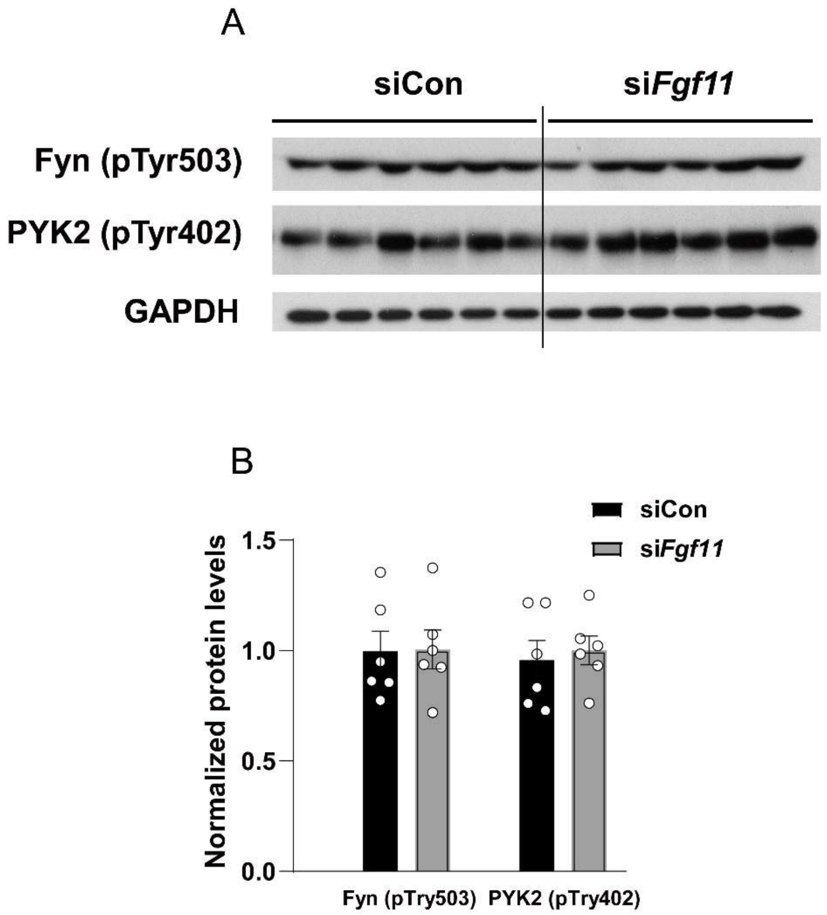

Supplementary Figure 7. Phosphorylation of upstream kinases of GSK3 after *Fgf11* knockdown in NPY/AgRP co-expressing cells. N41 cells were transfected with non-silencing control siRNA or si*Fgf11*, and phosphorylation of Fyn (Tyr503) and PYK2 (Tyr402) was examined by western blot analysis. Phosphoprotein levels were normalized to that of GAPDH. n = 6; n.s., not significant versus control.
